# The Use of International Classification of Diseases Codes to Identify Patients with Pancreatitis: A Systematic Review and Meta-analysis of Diagnostic Accuracy Studies

**DOI:** 10.1038/s41424-018-0060-1

**Published:** 2018-10-04

**Authors:** Amy Y. Xiao, Marianne L. Tan, Maria N. Plana, Dhiraj Yadav, Javier Zamora, Maxim S. Petrov

**Affiliations:** 1 Department of Surgery, University of Auckland, Auckland, New Zealand; 2 CIBER Epidemiology and Public Health (CIBERESP), Universidad Francisco de Vitoria, Madrid, Spain; 3 Division of Gastroenterology, Hepatology & Nutrition, University of Pittsburgh Medical Center, Pittsburgh, USA; 4 Barts and the London School of Medicine and Dentistry, Queen Mary University of London, London, UK

## Abstract

**Background:**

Hospital discharge codes are increasingly used in gastroenterology research, but their accuracy in the setting of acute pancreatitis (AP) and chronic pancreatitis (CP), one of the most frequent digestive diseases, has never been assessed systematically. The aim was to conduct a systematic literature review and determine accuracy of diagnostic codes for AP and CP, as well as the effect of covariates.

**Methods:**

Three databases (Pubmed, EMBASE and Scopus) were searched by two independent reviewers for relevant studies that used International Classification of Disease (ICD) codes. Summary estimates of sensitivity, specificity and positive predictive value were obtained from bivariate random-effects regression models. Sensitivity and subgroup analyses according to recurrence of AP and age of the study population were performed.

**Results:**

A total of 24 cohorts encompassing 18,106 patients were included. The pooled estimates of sensitivity and specificity of ICD codes for AP were 0.85 and 0.96, respectively. The pooled estimates of sensitivity and specificity of ICD codes for CP were 0.75 and 0.94, respectively. The positive predictive value of ICD codes was 0.71 for either AP or CP. It increased to 0.78 when applied to incident episode of AP only. The positive predictive value decreased to 0.68 when the ICD codes were applied to paediatric patients.

**Conclusion:**

Nearly three out of ten patients are misidentified as having either AP or CP with the indiscriminate use of ICD codes. Limiting the use of ICD codes to adult patients with incident episode of AP may improve identification of patients with pancreatitis in administrative databases.

## Introduction

Advancements in information technology have revolutionised the way individual patient data are collected and processed^1^. Increasingly, more simultaneous documentation and execution has allowed large amounts of data to be amassed in a short time^2^—a phenomenon that has been penned 'big data'. 'Big data' is defined by characteristics of large variety of sources, volume and velocity^3^. In the health industry, these sources can vary from regional databases of electronic health records and cancer registries to individual smartphone monitoring of sleep and diet^3^. Digitalisation has enabled practical and low-cost accessibility of ‘big data’, and one example of it is the use of administrative diagnostic codes. Diagnostic coding is now used ubiquitously, including application for the purpose of research^1^. Increasingly, larger cohorts are required to produce more generalisable results and distil out trends from background error^4^. Diagnostic codes are a practical method to achieve these goals^1^ and, therefore they have become engrained in medical research in general and gastroenterology research in particular^4^.

Pancreatitis poses a significant burden to health systems^5^, at least in part because there are still obstacles to accurate diagnosis of pancreatitis. Chronic pancreatitis (CP) has no universally accepted diagnostic criteria^6^. The Atlanta criteria to diagnose acute pancreatitis (AP) ^7^ offer a composite definition that is based on the presence of two out of the three domains (clinical, laboratory and radiological). Each pair of domains can have different diagnostic accuracy, and it is conceivable that individual doctors may favour one combination over another. Further, there is high variability in the reported positive predictive value of diagnostic coding in AP^8,9^. This not only has implications for the studies that rely on diagnostic coding, but also suggests possible overdiagnosing of AP. Further, inflated estimates of burden of AP may lead to excessive cost allocation, unnecessary procedures and may deflate estimates of mortality^10^.

The aim of this study was to conduct a systematic literature review of cohort studies to assess the accuracy of diagnostic codes for AP and CP and investigate the effect of covariates.

## Methods

### Search strategy

Three electronic databases (Pubmed, EMBASE and Scopus) were used to search for articles from the earliest available date until February 1, 2016. The Pubmed and EMBASE search strategy contained three sets of terms and the Scopus search strategy contained four sets. The Boolean operator 'AND' was used between the sets whereas the operator 'OR' was used within each set. For Pubmed, the first set contained “Drug prescriptions”, “Insurance, Health”, “Databases as topic”, “Clinical coding”, “Registries”, “Hospitalisation”, “International Classification of Disease” and “ICD”. The second set contained “Validation Studies as topic”, “Epidemiologic Research Design”, “Algorithm” and “Pancreatitis/epidemiology”. The third set contained “Pancreatitis”. These were all MeSH terms, except for “ICD”. For EMBASE, the terms were searched by subject heading and exploded where possible. The first set contained the exploded terms of “Health Services Research”, “Medical Records”, “International Classification of Disease”, “Prescriptions”, “Hospital Discharge”, “Billing and Claims” and “Coding” and the terms searched by keyword “Health Information”, “Surveillance”, “Administrative Data”, “Code$” and “ICD$”. The second set contained the exploded terms of “Validity”, “Validation Study” and “Algorithm” and the terms searched by keyword “Case Definition”, “Sensitivity”, “Specificity”, “Positive Predictive Value” and “Negative Predictive Value”. The third set contained the exploded term “Pancreatitis”. For Scopus, the first set contained “Prescription”, “Medical Records”, “Insurance Claim”, “Registries”, “Database” and “Hospital Discharge”. The second set contained “International Classification of Disease”, “ICD*”, “Coding” and “Code*”. The third set contained “Case Definition”, “Sensitivity”, “Specificity”, “Positive Predictive Value” and “Negative Predictive Value”. The fourth set contained “Acute Pancreatitis”. The search was limited to articles in English.

### Inclusion criteria

Included studies required to have reported at least one measure of diagnostic accuracy (such as sensitivity, specificity, positive predictive value and negative predictive value) in the setting of AP and/or CP. The accuracy of codes according to either ICD-8 or ICD-9, or ICD-10 (or a combination of the above) had to be compared with an independent reference standard formulated by experts in the field. The ICD codes explored in this study were all subtypes of K85 and K86.0, 86.1 from ICD 10 CM and 577.0, 577.1 from ICD 8.9. Two independent reviewers (A.Y.X.) and (M.L.T.) screened for eligible studies and any discrepancies were discussed with the senior author (M.S.P.).

### Exclusion criteria

Studies were excluded if there was inadequate information on the coding provided or no independent reference standard used. Cases of post-ERCP pancreatitis or postpartum pancreatitis were excluded. Studies with a sample size of less than 25 were also excluded, as well as studies focused on a particular aetiology of AP or CP.

### Data extraction

Extraction was performed on the following variables: type of administrative code, coding position, number of cases identified by the administrative code, reference standard used, number of cases verified by reference code, positive predictive value, negative predictive value, sensitivity and specificity. Positive predictive value (PPV), negative predictive value, sensitivity and specificity were calculated if not reported in the primary article and required data were available. Positive and negative likelihood ratios, as well as diagnostic odds ratios, were calculated for each study if adequate information was available. Paediatric and first episode of acute pancreatitis cases were also recorded.

### Quality assessment

The QUADAS (Quality Assessment of Diagnostic Accuracy Studies) tool^11^ was used to assess the methodological quality of the included studies based on a total of 14 items.

### Statistical methods

For studies in which it was possible to extract information on all four cells of the 2 × 2 table, sensitivity and specificity were estimated with 95% confidence intervals (CI). A bivariate random-effects regression model was fitted to obtain a summary receiver operating characteristic (SROC) curve and the corresponding area under the curve in order to take the potential trade-off between sensitivity and specificity explicitly into consideration and incorporate this negative correlation into the analysis^12^. Positive predictive values were calculated for all studies included. Mean PPV was obtained using a random-effects logistic regression. Sensitivity, specificity and PPV were represented graphically using the corresponding forest plots to investigate heterogeneity. Heterogeneity among studies was quantified with the variance of the logit of accuracy indices as estimated by the bivariate model, tau^2^ and I^2^ statistics. The minimum number of studies required to calculate heterogeneity was two. We selected a priori the following factors as potential sources of heterogeneity: ICD version, coding position, reference standard, recurrence of acute pancreatitis and age group of the patients. If the number of studies was sufficient, we investigated heterogeneity by adding covariate terms to the bivariate model to assess the effect of a covariate on accuracy. Statistical analyses were conducted using the Metandi and Metaprop_one programs for the STATA software^13^.

## Results

### Characteristics of the included studies

**Fig. 1:**
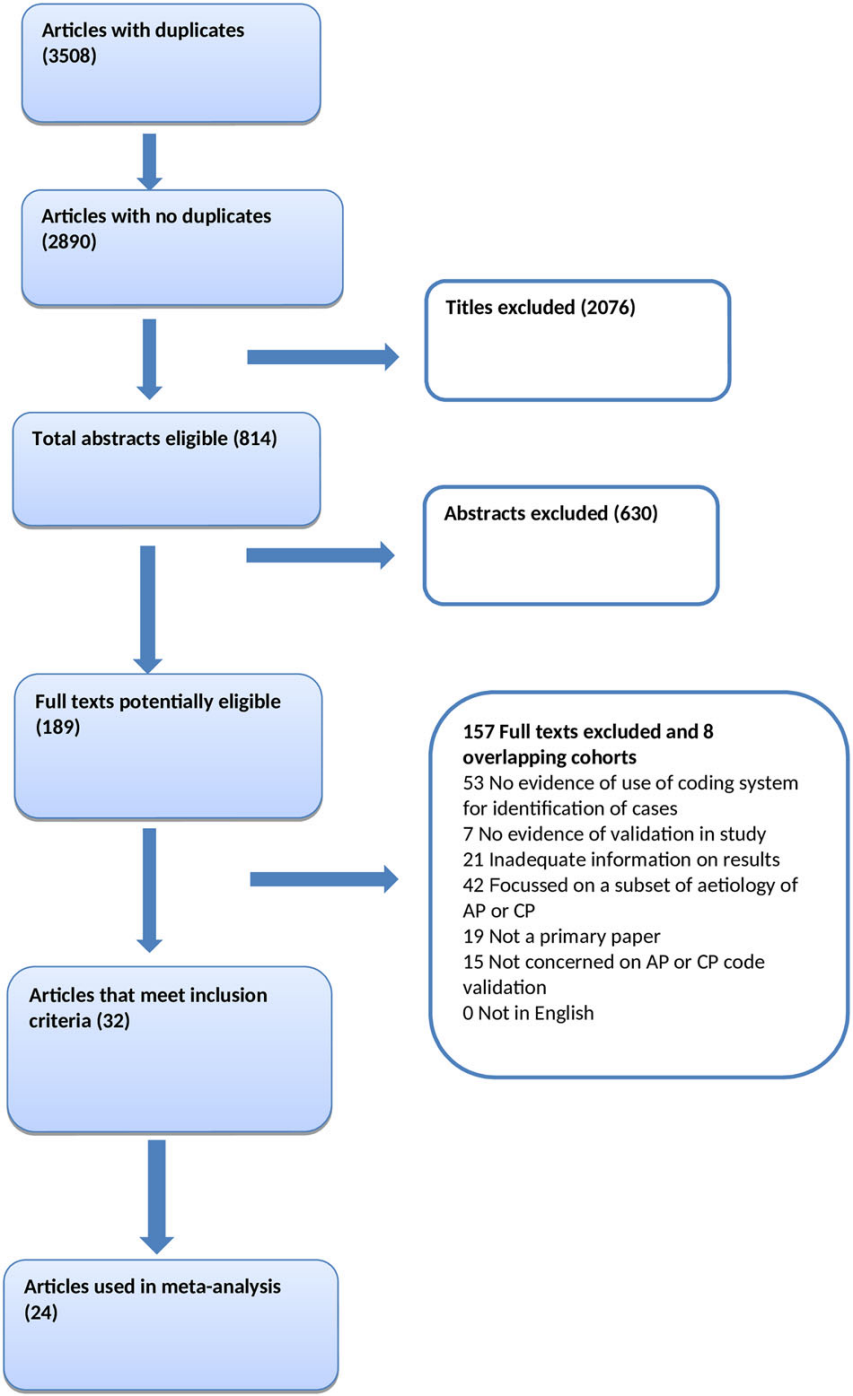
Flow chart of the study selection process

A total of 24 studies were included in the final analysis (Fig. 1). Baseline characteristics of all the included cohorts are shown in Table 1. A total of 21 cohorts investigated AP^8,14–33^ and seven cohorts—CP^15,18,20,21,34–36^. In AP, two cohorts used ICD-8, 15—ICD-9 and five—ICD-10. In CP, two cohorts used ICD-8, five—ICD-9 and two—ICD-10. The total number of individuals in the source population was 18,106 (6858 with AP; 1927 with CP; 8537 with diseases other than AP and 784 with diseases other than CP). The total number of validated cases was 7464 (5668 with AP and 1796 with CP). The median study period was 3 years with an interquartile range of 2 to 10 years. Methodological quality of the included studies is presented in Tables 2 and 3.

**Table 1:** Characteristics of the included cohorts

Author	Country	Database	Study period	Patient population	Coding system	Coding position	Reference standard	Total (n)	Cases coded(n)	PPV	NPV	Sensitivity	Specificity	Likelihood ratios positive/ negative
*Acute pancreatitis*														
Eland et al.^14^	Netherlands	National Information System on Hospital Care	1985, 1990, 1995	Adult	ICD-9	Primary	Non-Atlanta criteria	101	101	0.82	–	–	–	–
Porta et al.^15^	Spain	PANKRAS II Study	1992–1995	Adult	ICD-9	Primary	Not reported	600	37	0.54	0.96	0.50	0.97	17.81/0.47
Chwistek et al.^16^	United Kingdom	Bridgeport Hospital	1994– 1996	Adult	–	Primary or secondary	Non-Atlanta criteria	145	145	0.85	–	–	–	–
Floyd et al.^17^	Denmark	Hospital Discharge Registry	1981–2000	Adult	ICD-8, ICD-10	Primary or secondary	Not reported	99	99	0.82	–	–	–	–
Quraishi et al.^8^	United States	Henry Ford Health System	1998– 2003	Adult	ICD-9	Primary or secondary	Non-Atlanta criteria	1393	128	0.22	1.00	1.00	0.93	13.65/0
Yadav et al.^18^	United States	Veterans Outpatient Detoxification Programme	2002–2003	Adult	ICD-9	Primary or secondary	Non-Atlanta criteria	50	50	0.32	–	–	–	–
Kandula et al.^19^	United States	Children’s Hospital of Pittsburgh	1995–2004	Paediatric	ICD-9	Primary or secondary	Non-Atlanta criteria	109	109	0.80	–	–	–	–
Spanier et al.^20^	Netherlands	Academic Medical Centre	2002–2003	Adult	ICD-9	Primary or secondary	Non-Atlanta criteria	523	112	0.78	0.89	0.65	0.94	10.2/0.37
Nojgaard et al.^21^	Denmark	Hvidovre Hospital Admissions	1983, 1994, 2005	Adult	ICD-8, ICD-10	Primary or secondary	Non-Atlanta criteria	165	165	0.64	–	–	–	–
Dore et al.^22^	United States	Normative Health Information Database	2005–2007	Adult	ICD-9	Primary or secondary	Atlanta criteria	585	585	0.50	–	–	–	–
Omdal et al.^23^	Norway	Haukeland University	1996–2006	Adult	ICD-9, ICD-10	Primary or secondary	Non-Atlanta criteria	724	724	0.78	–	–	–	–
Razavi et al.^24^	Sweden	Swedish National Patient Registry	1998–2007	Adult	ICD-10	Primary or secondary	Atlanta criteria	603	530	0.83	0.69	0.95	0.36	1.49/0.14
Ma et al.^25^	United States	Yale New Haven Children’s Hospital	1994– 2007	Paediatric	ICD-9	Primary or secondary	Non-Atlanta criteria	548	548	0.50	–	–	–	–
Edwards et al.^26^	United Kingdom	Derriford Hospital Emergency	2009– 2010	Adult	Not reported	Primary or secondary	Non-Atlanta criteria	231	231	0.29	–	–	–	–
Shen et al.^27^	Taiwan	National Health Insurance Research Database	2006–2008	Adult	ICD-9	Primary or secondary	Atlanta criteria	50	50	0.90	–	–	–	–
Saligram et al.^28^	United States	University of Pittsburgh Medical Centre	2000, 2002, 2005	Adult	ICD-9	Primary	Atlanta criteria	803	401	0.77	0.97	0.97	0.81	5.11/0.04
Podugu et al.^29^	United States	Cleveland Clinic	2010–2011	Adult and paediatric	ICD-9	Primary	Atlanta criteria	480	480	0.68	–	–	–	–
Wu et al.^30^	United States	Kaiser Permanente Southern California	2006–2012	Adult	ICD-9	Primary	Not reported	100	100	0.55	–	–	–	–
Shinagare et al.^31^	United States	Brigham and Women’s Hospital	2012– 2013	Adult	ICD-9	Primary or secondary	Atlanta criteria	115	115	0.89	–	–	–	–
Bertilsson et al.^32^	Sweden	Skane University Hospital	2003–2012	Adult	ICD-10	Primary or secondary	Atlanta criteria	2112	2112	0.87	–	–	–	–
Yang et al.^33^	United States	Mayo Clinic	2011–2013	Adult	ICD-9	Primary	Not reported	6096	273	1.00	0.94	0.44	1.00	Not calculable/ 0.56
*Chronic pancreatitis*														
Porta et al.^15^	Spain	PANKRAS II Study	1992–1995	Adult	ICD-9	Primary	Non-standard	600	89	0.87	0.93	0.68	0.98	32.61/0.33
Bagul et al.^34^	United Kingdom	Manchester Royal Infirmary	1993	Adult	ICD-9	Primary or secondary	Non-standard	45	45	0.91	–	–	–	–
Yadav et al.^18^	United States	Veterans Outpatient Detoxification Programme	2002–2003	Adult	ICD-9	Primary or secondary	Ammann’s criteria	15	15	0.07	–	–	–	–
Spanier et al.^20^	Netherlands	Academic Medical Centre	2002–2003	Adult	ICD-9	Primary or secondary	Non-standard	523	250	0.84	0.79	0.79	0.84	4.93/0.25
Joergensen et al.^35^	Denmark	Danish National Registry	1977– 2004	Adult	ICD-8, ICD-10	Primary or secondary	Mayo Clinic diagnostic scoring system	719	719	0.81	–	–	–	–
Nojgaard et al.^21^	Denmark	Hvidovre Hospital Admissions	1983, 1994, 2005	Adult	ICD-8, ICD-10	Primary or secondary	Mayo Clinic diagnostic scoring system	185	185	0.72	–	–	–	–
Reddy et al.^36^	United States	University of Michigan Health Service Database	2005– 2008	Adult	ICD-9	Primary or secondary	Mayo Clinic diagnostic scoring system ; Ammann’s criteria; Japanese Pancreas Society criteria	1343	1343	0.49	–	–	–	–

**Table 2:** QUADAS analysis of the acute pancreatitis cohorts

Study ID	1	2	3	4	5	6	7	8	9	10	11	12	13	14
Eland et al.^14^	Y	Y	N	Y	Y	Y	Y	Y	Y	Y	N	Y	Y	Y
Porta et al.^15^	Y	Y	U	Y	Y	Y	U	Y	N	Y	Y	Y	Y	Y
Chwistek et al.^16^	Y	Y	Y	Y	Y	Y	Y	N	Y	Y	N	Y	Y	Y
Floyd et al.^17^	Y	Y	N	Y	Y	Y	Y	Y	Y	Y	N	Y	Y	Y
Quraishi et al.^8^	N	Y	Y	Y	Y	Y	Y	Y	Y	Y	N	Y	Y	Y
Yadav et al.^18^	N	Y	Y	Y	Y	Y	Y	Y	Y	Y	Y	Y	Y	Y
Kandula et al.^19^	N	Y	Y	Y	Y	Y	Y	Y	Y	Y	N	Y	Y	Y
Spanier et al.^20^	Y	Y	Y	Y	Y	Y	Y	Y	Y	Y	N	Y	Y	Y
Norjgaard et al.^21^	Y	Y	N	Y	Y	Y	Y	Y	Y	Y	N	Y	Y	Y
Dore et al.^22^	N	Y	Y	Y	Y	Y	Y	Y	Y	Y	N	Y	Y	Y
Omdal et al.^23^	Y	Y	Y	Y	Y	Y	Y	Y	Y	Y	N	Y	Y	Y
Razavi et al.^24^	Y	Y	Y	Y	Y	Y	Y	Y	Y	Y	N	Y	Y	Y
Ma et al.^25^	N	Y	Y	Y	Y	Y	Y	Y	Y	Y	N	Y	Y	Y
Edwards et al.^26^	Y	Y	N	Y	Y	Y	Y	N	Y	Y	N	Y	Y	Y
Shen et al.^27^	Y	Y	Y	Y	Y	Y	Y	Y	Y	Y	N	Y	Y	Y
Saligram et al.^28^	Y	Y	Y	Y	Y	Y	Y	Y	Y	Y	N	Y	Y	Y
Podugu et al.^29^	Y	Y	Y	Y	Y	Y	Y	Y	Y	Y	N	Y	Y	Y
Wu et al.^30^	Y	Y	U	Y	Y	Y	U	Y	N	Y	N	Y	Y	Y
Shinagare et al.^31^	Y	Y	Y	Y	Y	Y	Y	Y	Y	Y	N	Y	Y	Y
Bertilsson et al.^32^	Y	Y	Y	Y	Y	Y	Y	Y	Y	Y	N	Y	Y	Y
Yang et al.^33^	Y	Y	U	Y	Y	Y	U	Y	N	Y	N	Y	Y	Y

**Table 3:** QUADAS analysis of the chronic pancreatitis cohorts

	1	2	3	4	5	6	7	8	9	10	11	12	13	14
Porta et al.^15^	Y	Y	N	N	Y	Y	U	Y	Y	Y	Y	N	Y	Y
Bagul et al.^34^	Y	Y	U	N	Y	Y	U	Y	N	Y	N	N	Y	Y
Yadav et al.^18^	N	Y	Y	N	Y	Y	Y	Y	Y	Y	Y	N	Y	Y
Spanier et al.^20^	Y	Y	Y	N	Y	Y	Y	Y	Y	Y	N	N	Y	Y
Joergensen et al.^35^	N	Y	Y	N	Y	Y	Y	Y	Y	Y	N	N	Y	Y
Norjgaard et al.^21^	Y	Y	Y	N	Y	Y	Y	Y	Y	Y	N	N	Y	Y
Reddy et al.^36^	Y	Y	Y	N	Y	Y	Y	Y	Y	Y	N	N	Y	Y

### Studies in acute pancreatitis

**Fig. 2:**
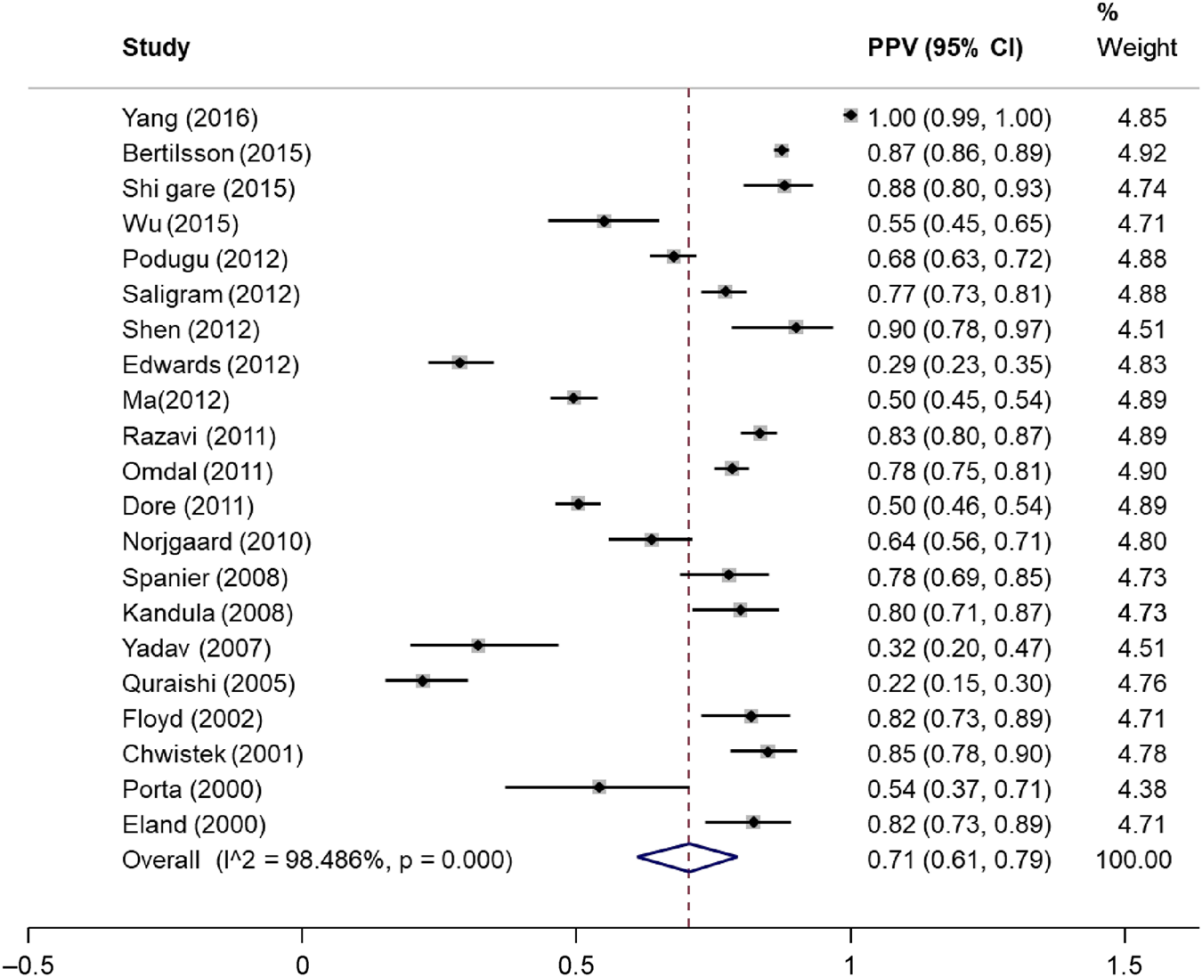
Pooled positive predictive value of ICD codes in identifying patients with acute pancreatitis

**Fig. 3:**
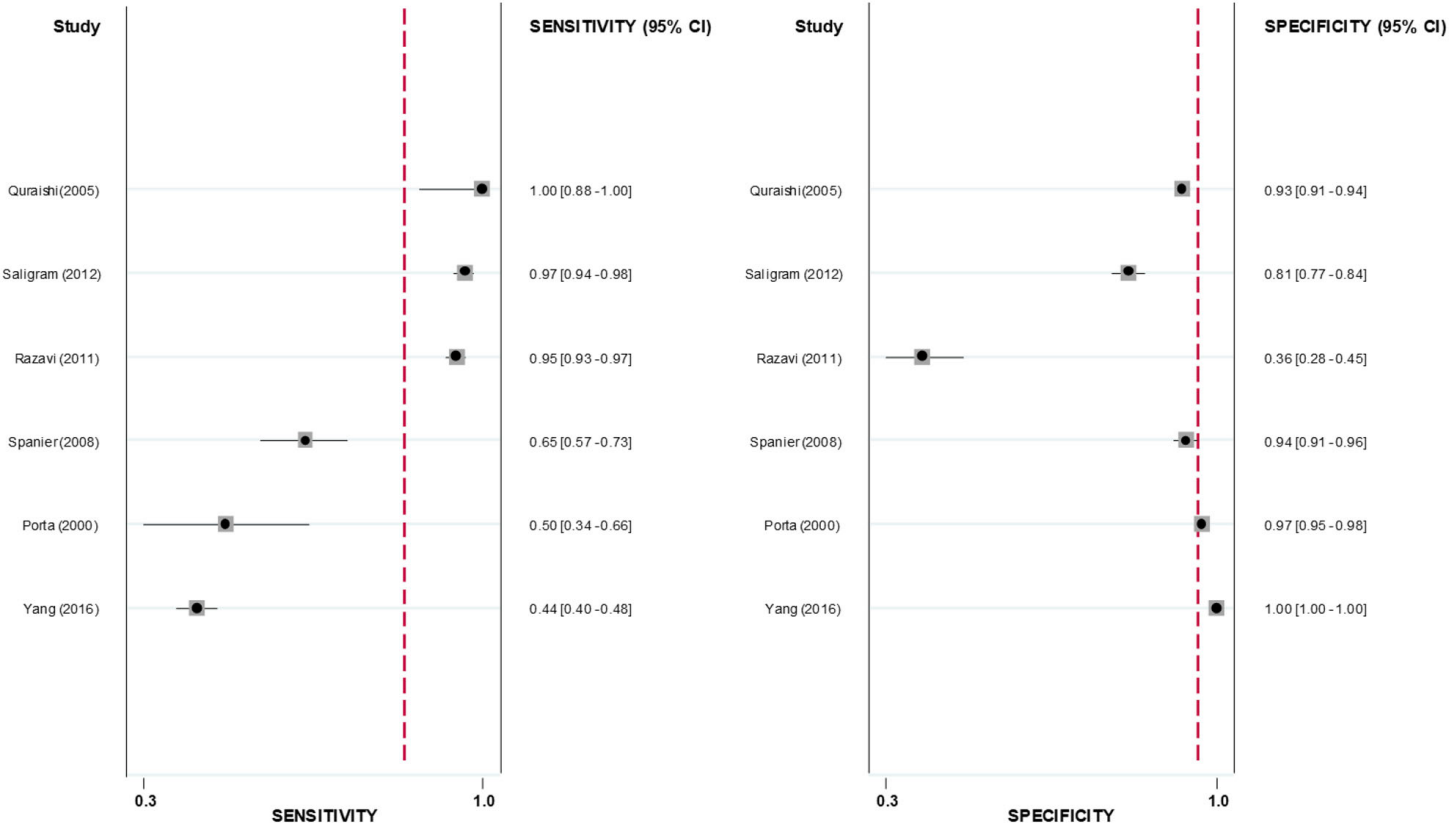
Pooled sensitivity and specificity of ICD codes in identifying patients with acute pancreatitis

**Fig. 4:**
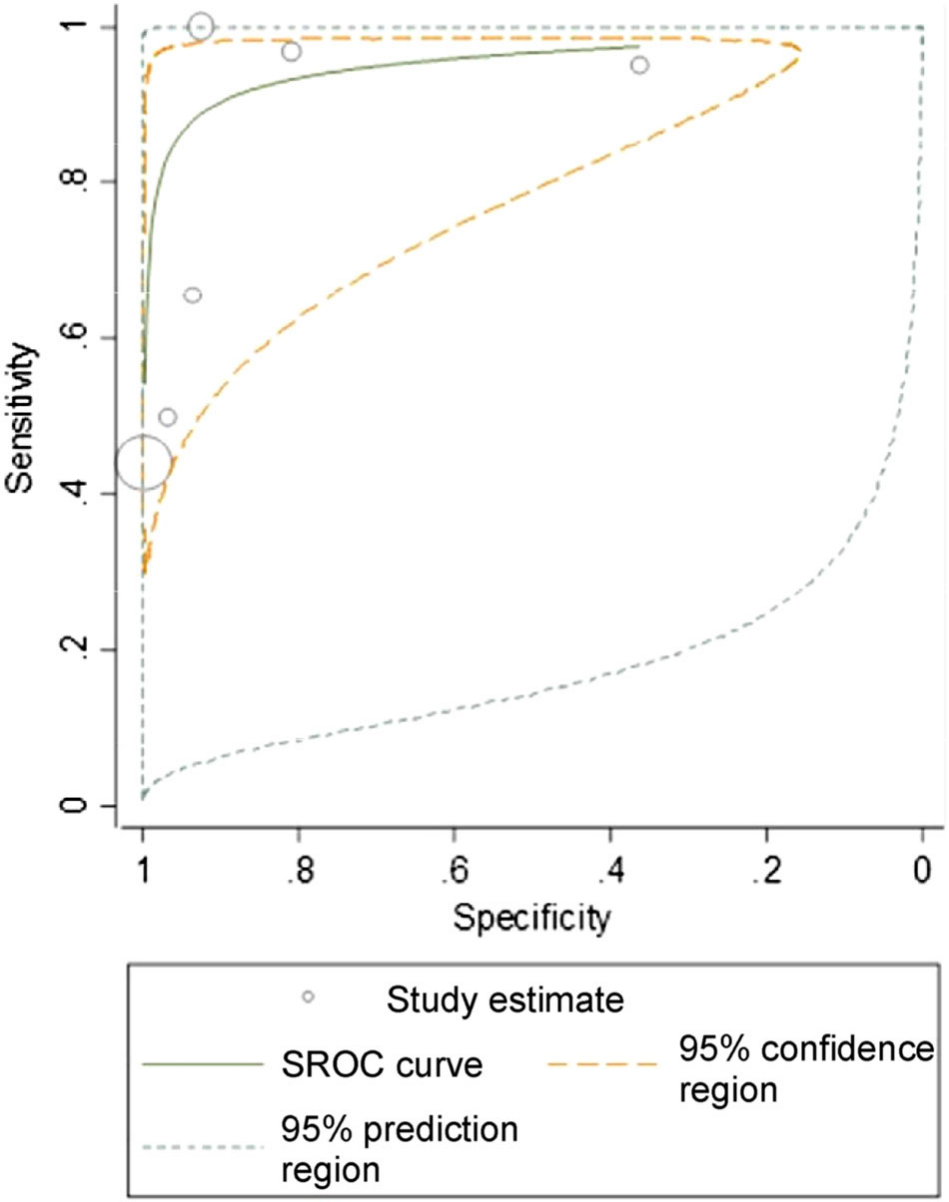
Summary receiver operating characteristic (SROC) curve of sensitivity and specificity of ICD codes in identifying patients with acute pancreatitis

A total of 21 cohorts reported on the PPV of ICD codes for AP. The crude pooled PPV was 0.71 (95% CI 0.61–0.79; *p* < 0.0001; I^2^ = 98.5%) (Fig. 2). Six cohorts (10,018 participants) reported on sensitivity, specificity, positive likelihood ratio, negative likelihood ratio and diagnostic odds ratio. The crude pooled sensitivity and specificity were 0.85 (95% CI 0.59–0.96) and 0.96 (95% CI 0.65–1.00), respectively (Fig. 3). The crude pooled positive likelihood ratio, negative likelihood ratio and diagnostic odds ratio were 21.6 (95% CI 2.1–223.7), 0.2 (95% CI 0.1–0.5) and 137.8 (95% CI 19.0–1001.4), respectively. The SROC curve produced an area under the curve of 0.95 (95% CI 0.56–1.00) (Fig. 4).

The subgroup analysis according to the versions of ICD included 10,809 participants from 14 cohorts that used ICD-9 alone, as well as 2855 participants from three cohorts that used ICD-10 alone. The PPV for ICD-9 codes was 0.69 (95% CI 0.55–0.81; Tau^2^ = 0.271; I^2^ = 98.3%), whereas the one for ICD-10 was 0.79 (95% CI 0.69–0.88; Tau^2^ = 0.042; *p* = 0.189; I^2^ = 95.7%), *p* = 0.189. The subgroup of adult patients only included 14,938 participants from 19 cohorts and yielded a PPV of 0.71 (95% CI 0.61–0.80; Tau^2^ = 0.207; I^2^ = 98.4%), whereas the subgroup of paediatric patients only included 694 participants from three cohorts and yielded a PPV of 0.68 (95% CI 0.44–0.88; Tau^2^ = 0.173; I^2^ = 95.5%), *p* = 0.826. The subgroup analysis according to definitions of AP showed that studies that used the Atlanta definition as the reference standard (4163 participants from seven cohorts) yielded a PPV of 0.79 (95% CI 0.67–0.88; Tau^2^ = 0.123; I^2^ = 98.4%). The remaining 14 cohorts (10,725 participants) used a reference standard other than the Atlanta definition and yielded a PPV of 0.66 (95% CI 0.51–0.80; Tau^2^ = 0.337; I^2^ = 98.5%). The subgroup analysis according to coding position included 9881 participants from seven cohorts with primary coding position and yielded the PPV of 0.75 (95% CI 0.59–0.88; Tau^2^ = 0.207; *p* = 0.596; I^2^ = 98.3%). The PPV for primary or secondary coding position was 0.81 (95% CI 0.773–0.837; Tau^2^ = 0.003; *p* = 0.596; I^2^ = 48.4%). The sensitivity analysis constrained to incident episode of AP only was based on five cohorts (1718 patients) and yielded a PPV of 0.78 (95% CI 0.70–0.85; Tau^2^ = 0.033; *p* = 0.209; I^2^ = 87.1%).

**Fig. 5:**
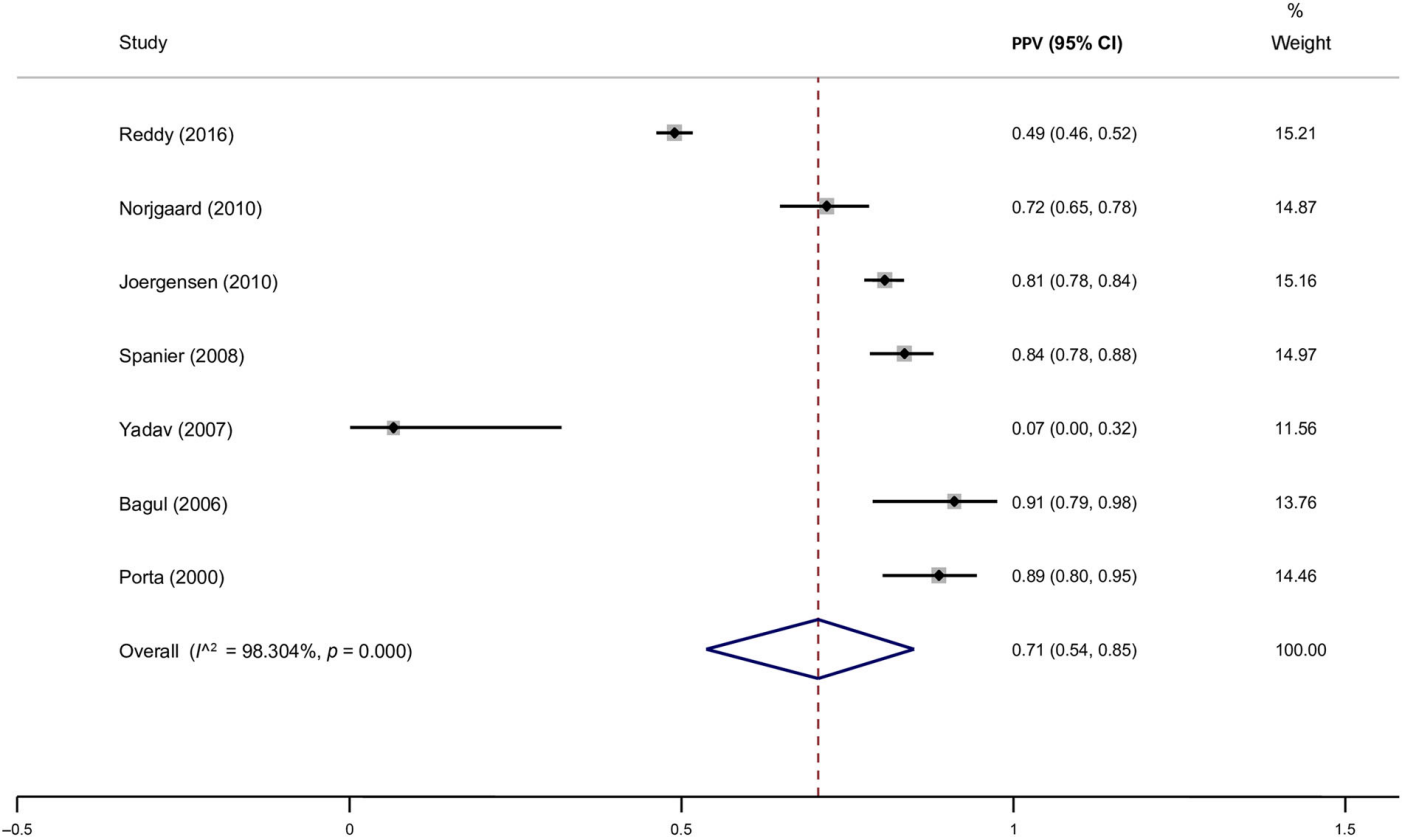
Pooled positive predictive value of ICD codes in identifying patients with chronic pancreatitis

### Studies in chronic pancreatitis

A total of seven cohorts reported on the PPV of ICD codes for CP. The crude PPV was 0.71 (95% CI 0.54–0.85; *p* < 0.0001; I^2^ = 98.3%) (Fig. 5). The sensitivity analysis constrained to ICD-9 version only included five cohorts (2526 participants) and yielded a PPV of 0.67 (95% CI 0.42–0.89; Tau^2^ = 0.320; *p* = 0.301; I^2^ = 98.2%). Only two cohorts (1123 participants) reported on sensitivity and specificity, which yielded pooled values of 0.75 (95% 0.71–0.80) and 0.94 (95% CI 0.93–0.96), respectively. There was an insufficient number of cohorts to perform other pre-specified analyses.

## Discussion

This is the first systematic literature review and meta-analysis to report on pooled estimates of accuracy of the ICD codes for identifying patients with AP and CP. The pooled PPV for AP in the present study was 0.71. Systematic literature reviews on accuracy of ICD codes in other acute conditions found pooled estimates of PPV to be 0.82 in ischaemic stroke^37^, 0.92 in myocardial infarction^38^ and 0.93 in subarachnoid haemorrhage^37^. Similarly, the pooled PPV for CP in the present study was 0.71. Systematic literature reviews on accuracy of ICD codes in other chronic conditions found pooled estimates of PPV to be 0.87 in heart failure^39^ and 0.89 in depression^40^. Taken together, the above findings suggest that accuracy of ICD codes in identifying patients with AP and CP is, in general, inferior to other acute and chronic conditions.

A series of pre-specified analyses showed that higher PPV of ICD codes for AP is reached when ICD-10, as opposed to ICD-9, is used; when the codes are applied to incident episode of AP as opposed to recurrent AP and when cases are validated with the use of Atlanta definition. Specifically, the subgroup analysis according to versions of ICD showed that ICD-10 codes yield a 10% higher PPV than that of ICD-9 codes, and this is likely a reflection of improvements in diagnostic methods^41^. ICD-10 also requires the input of aetiology of AP^24^, which would require more confidence in the diagnosis. It is assuring that ICD-10 is now the most commonly used version of ICD^42^, and improvement of PPV of the ICD codes for AP is expected in the future. The sensitivity analysis limited to cases of only incident episode of AP showed a 7% higher PPV in comparison with the overall AP cohort. This suggests that misdiagnosis may occur when re-admitted patients with previous pancreatitis are assumed to have another episode of pancreatitis^43^. Analysis of cases validated with the use of Atlanta definition yielded the PPV of 0.79. Although this is an improvement in comparison with the overall estimate, it is worrying that 21% cases are diagnosed with AP when, in fact, they do not have it. The other noteworthy finding is that the PPV of diagnostic codes is lower in children, with a PPV of just 0.68. Of note, our study did not find PPV of AP to be improved in the subgroup analysis of primary coding position (0.75) in comparison to primary or secondary coding position (0.81). The value of PPV in primary or secondary coding positions may be higher than that of primary coding position alone because the diagnosis of AP was more confidently made when in conjunction with another related diagnosis, such as cholelithiasis.

Given the generally moderate PPV values of ICD codes for AP and CP, the main clinical implication of the present study is that overdiagnosing of pancreatitis is frequent. Patients with a previous history of AP are likely to be re-admitted with the coding of an episode of AP again^44^. This episode may be a continuation of a previous inadequately treated episode or it could be a different pathology at all^43^. One previous code of AP predisposes a patient to more likely receive future pancreatitis diagnostic codes^28^. Advances in serum testing have allowed detection of more mild cases of AP, but has also led to more overdiagnosing^45^. The diagnosis of early CP remains a significant challenge. One component of the diagnostic criteria for CP is histology, which is often unavailable at the time of coding^46^. The diagnosis, thus, becomes predominantly based on imaging modalities^46^.

The main immediate implication for research is that a correction factor may need to be employed to estimate accurately the real burden of pancreatitis in the studies that used ICD codes. Leong and colleagues suggested a formula that uses specificity and sensitivity to give a corrected prevalence^47^. This formula may be useful for correcting the prevalence of CP rather than AP. Ley and colleagues, as well as Esposito and colelagues, proposed the use of PPV itself as a correction factor for incidence and this would be more appropriate for AP^48,49^. While development of more accurate diagnostic codes is anticipated in the future, the pooled PPV value of 0.71 in the present study can be used to derive corrected incidence of AP in the existing literature. There are also other ways to improve on accuracy of epidemiological estimates in the field of Pancreatology. Participants can be recruited in future studies by searching for the unique patient rather than for the episode^28^. Exclusion of patients with a previous pancreatitis diagnosis can increase the PPV as these cases tend to have a higher chance of a misdiagnosed readmission^28^. The requirement of elevated pancreatic enzyme levels above a three-time threshold, as suggested by current guidelines, may further increase the accuracy of ICD codes^28^.

The limitations of the present study need to be acknowledged. First, the included studies came from different countries and from hospitals of various size, which may have contributed to heterogeneity. Second, the validation criteria used in the primary studies were not standardised. Third, PPV as a measure of diagnostic accuracy is affected by disease prevalence^50^. Given that CP is a much less common disease than AP, PPV for CP may have been low due to its relatively low prevalence^5^. Last, inclusion of primary studies was restricted to English, and this may have led to a language bias.

In conclusion, the overall diagnostic accuracy of ICD codes for pancreatitis is suboptimal. It is higher when the codes are applied to incident episode of AP and to adults, as well as when ICD-10 is used. The correction factor of 0.71 can be used to estimate accurately the burden of AP in studies using administrative databases. In the future, new diagnostic criteria may need to be developed for patients with recurrent AP and CP.
