# Correction: The Use of International Classification of Diseases Codes to Identify Patients with Pancreatitis: A Systematic Review and Meta-analysis of Diagnostic Accuracy Studies

**DOI:** 10.1038/s41424-018-0078-4

**Published:** 2018-11-21

**Authors:** Amy Y. Xiao, Marianne L. Tan, Maria N. Plana, Dhiraj Yadav, Javier Zamora, Maxim S. Petrov

**Affiliations:** 1 Department of Surgery, University of Auckland, Auckland, New Zealand; 2 CIBER Epidemiology and Public Health (CIBERESP), Universidad Francisco de Vitoria, Madrid, Spain; 3 Division of Gastroenterology, Hepatology & Nutrition, University of Pittsburgh Medical Center, Pittsburgh, USA; 4 Barts and the London School of Medicine and Dentistry, Queen Mary University of London, London, UK

Correction to: *Clinical and Translational Gastroenterology*; 10.1038/s41424-018-0060-1; published online 04 October 2018.

The original version of this Article presented incorrect information for citation 50. This has now been corrected in both the PDF and HTML versions of the Article.

